# The fragile dialogue: communication barriers, authority and adaptive strategies in NICU parent-healthcare worker relationships

**DOI:** 10.3389/fsoc.2025.1683833

**Published:** 2025-11-26

**Authors:** Alessandra Decataldo, Giacomo Lauritano, Federico Paleardi

**Affiliations:** Department of Sociology and Social Research, University of Milan-Bicocca, Milan, Italy

**Keywords:** preterm parents, NICU communication, healthcare staff-parents interactions, health communication, qualitative research

## Abstract

**Introduction:**

Preterm birth profoundly impacts both the infant’s health and the family’s psychosocial well-being. In NICUs, communication between professionals and parents unfolds in contexts of high emotional stress, technical complexity and structural power asymmetries. Whilst effective dialogue supports family well-being, some structural and contextual factors in the studied NICUs often prevent it. This study, part of the e-ParWelB project, examines healthcare staff’s perspectives on structural barriers, the role of digital technologies, and authority dynamics, especially strategies for managing high-uncertainty communication with preterm parents.

**Materials and methods:**

We conducted 76 semi-structured expert interviews with a maximum variation sample of NICU staff across four Italian hospitals. Focused ethnographic observations complemented interviews. Data were analysed using a concept-driven coding strategy in NVivo 15.

**Results:**

Barriers extend beyond language and ethnicity, including vertical (educational) and horizontal (disciplinary) gaps. Digital technologies increase parental assertiveness but also fuel misunderstandings, anxiety and mistrust. Parents’ peer group chats offer support but can amplify stress and conflict. Clinicians respond with varied, individualised strategies, especially pedagogical explanations and emotional support. In a landscape where their authority requires continual negotiation, they struggle to preserve their professional legitimacy whilst providing the best possible care for newborns and cultivating relationships with parents.

**Discussion:**

NICU communication is shaped by structural inequality, shifting authority and digital mediation. Healthcare staff broadly agree on an increased emphasis on relationships with parents compared to the past. Nonetheless, implicit and explicit challenges to professional authority often manifest in expectations that parents legitimise their involvement by demonstrating commitment through constant presence in the NICU and compliance with staff directives. Enhancing relational competence, embedding cultural mediation and institutionalising collaboration with parent associations could help reframe these dynamics into trust-based and inclusive forms of care, to the benefit of both families and healthcare workers.

## 1 Introduction

Preterm birth is a potentially distressing experience that affects not only the infant’s medical condition but also the emotional stability and everyday life of the family. These challenges are particularly evident during the critical period of hospitalisation in the Neonatal Intensive Care Unit (NICU) and the delicate transition back home. A recent literature review (Fazio et al., 2022) points out how a positive parent-healthcare worker’s communication can greatly improve families’ well-being during this critical period. However, the learning of targeted communication skills is still not considered a relevant part of most healthcare workers’ training programs.

The relationships between healthcare professionals and parents, particularly in critical medical settings like NICUs, are inherently shaped by power asymmetry, and this is a core issue in the sociology of medicine (Gabe and Monaghan, 2022). Classical sociology has criticised medical institutions as sites where professional authority has marginalised the patients’ or parents’ agency (Goffman, 1961): these dynamics underline the tensions between the legitimacy of science and medical authority and the rights of patients. Recent times have seen a growing demand for patient dignity, autonomy, and meaningful participation in medical decision-making, whilst medical professionals, steeped in clinical expertise and institutional protocols, sometimes perceive these expectations as undermining their authority and disrupting established care practices (Henderson, 2003; Nimmon and Stenfors-Hayes, 2016; Stevens et al., 2021).

Similar tensions emerge in NICUs, where preterm parents could be defined as second-order patients (Mesman, 2014) since they wear hybrid roles as they are not patients themselves but are much more than mere visitors. They are expected to trust medical expertise yet increasingly demand involvement and shared decision-making in the care process; these power negotiations are further complicated by structural inequalities that impact the capacity of parents to advocate for empowerment. Ultimately, these dynamics have profound consequences, not only shaping parental well-being but also reinforcing systemic barriers that make the NICU experience even more challenging for vulnerable families.

This contribution explores the complex communication dynamics in the NICUs from the healthcare staff perspective. It sheds light on the ways in which healthcare professionals communicate with preterm parents, the obstacles they face, their experience and the relational arrangements developed when engaging with families. This study is part of a larger research titled e-ParWelB (website: https://parwelb.unimib.it/), which aims to construct an integrated socio-psychological framework by combining social science methodologies, digital health tools, and targeted psychological support for parents of preterm infants. The sociological strand of the overall project adopts a mixed-methods approach, which aims to give voice to preterm parents, their experiences and to study the social determinants of their well-being during this impactful experience, but also to consider the point of view of the healthcare staff who work every day in the NICU. Thus, the project combines survey research targeting preterm parents, in-depth expert interviews with healthcare professionals and multi-sited ethnographic fieldwork conducted in NICUs and sub-NICUs across four Italian hospitals.

With this contribution, we address three main aims:

1. Analyse structural barriers, particularly linguistic divides, that hinder effective dialogue;
2. Examine how digital technologies, especially the Internet, shape health staff and parents’ interactions and how it reconfigures power dynamics in clinical communication, as parents leverage online information to negotiate authority or compensate for informational asymmetries (Evans, 2008);
3. Investigate communication management and the adaptive strategies deployed by clinicians to navigate these challenges, balancing their professional authority with the need to mitigate parental distress in high-uncertainty contexts.

The paper is structured as follows: the first section outlines the theoretical framework; the second presents the materials and methods; the third is dedicated to the results, organised around the study’s three research objectives; and the final section discusses the findings and offers concluding reflections.

## 2 Theoretical framework

Communication between parents and healthcare professionals in NICUs represents a critical dimension of the perinatal hospital experience. NICUs are high-intensity relational environments, marked by clinical and social vulnerabilities, where expert knowledge, emotional needs, and structural inequalities intersect. Medical sociology has long emphasised how healthcare institutions function as asymmetrical spaces of negotiation, in which expert knowledge tends to overdetermine the scope for patient participation or, in the case of NICUs, parental involvement (Gabe and Monaghan, 2022; Goffman, 1961; Turner, 1992).

Within this context, three main analytical axes emerge: structural barriers to communication, the impact of digital technologies on power dynamics, and the adaptive strategies enacted by professionals to manage relational complexity.

Structural barriers that hinder effective communication between healthcare professionals and parents in NICUs are numerous and often interrelated. These obstacles extend beyond ethnic or linguistic differences and encompass what Evans (2008) defines as symbolic fractures—disruptions generated by the gap between the technical-scientific language of medicine and the interpretive capacities of laypersons. This gap becomes particularly pronounced in NICU settings, where communication concerns highly complex clinical conditions, time-sensitive decisions and intense emotional burdens (Franck et al., 2017; Russo and Decataldo, 2024).

To these symbolic fractures, one must add structural asymmetries in knowledge and authority that manifest in both vertical and horizontal barriers to communication. Vertical barriers arise when there is a significant asymmetry in educational background and social status between physicians and parents. These barriers are particularly relevant when caregivers have low levels of formal education or limited health literacy, which hinders their ability to understand medical terminology, interpret prognostic information, or formulate questions that would enable meaningful dialogue. As Schillinger et al. (2003) highlight, low health literacy is associated with decreased patient engagement, less adherence to care plans, and reduced satisfaction with the clinical encounter. In NICUs, this dynamic may exacerbate parental stress and lead to misunderstandings that compromise shared decision-making (Mesman, 2020).

Moreover, power differentials, reinforced by institutional settings and professional hierarchies, can discourage parents from voicing concerns or doubts, particularly when they perceive the medical team as inaccessible or authoritative. This effect is often magnified among marginalised or lower-income populations who may already have a history of mistrust or disempowerment within healthcare systems (Gabe and Monaghan, 2022; Goffman, 1961; Turner, 1992). Even in the absence of formal educational disadvantages, horizontal barriers may emerge. These occur between physicians and parents who, whilst being highly educated, lack specific biomedical knowledge. As Montgomery (2006) points out, professional expertise constructs an “epistemic enclave” that is not easily accessible to those outside the medical community. The specialised vocabulary, implicit assumptions and procedural logics of neonatal care can render lay understanding difficult even for professionals in non-medical fields. These horizontal fractures are not necessarily rooted in deference but rather in cognitive misalignments—differences in interpretive frameworks, expectations and emotional stakes (Heritage and Maynard, 2006). Such dynamics can lead to miscommunication or superficial agreement, where parents appear to understand or consent but have not fully grasped the clinical implications (Arabiat et al., 2018).

Emotional barriers further complicate these interactions. Professionals must manage not only their emotional expressions, but also those of parents, within a clinical culture that often rewards emotional restraint and procedural detachment. As Mesman (2014) observes, healthcare staff assume the role of “emotional managers,” navigating parental fears, buffering distress, and modulating their responses to stabilise the emotional climate surrounding highly vulnerable families. Parents, however, enter NICUs in states of acute emotional fragility—experiencing fear, grief or guilt—which may be unintentionally invalidated or ignored by institutional norms (O'Rourke et al., 2019). When these emotions are not legitimised within the communicative exchange, mutual trust can be undermined.

Moreover, gender barriers persist in shaping expectations and roles within NICU communication. As shown in the literature on gender and healthcare, mothers are disproportionately positioned as primary caregivers and emotional mediators, whilst fathers often experience marginalisation or emotional invisibility (Arendell, 2000; Jackson and Mannix, 2004; Lundqvist et al., 2007). These dynamics reinforce existing gender hierarchies in caregiving and professional authority and contribute to the unequal distribution of emotional burden within the family unit.

In this context, the notion of social fluency—i.e. the ability to understand and actively participate in expert domains—emerges as an informal yet powerful criterion of exclusion (Evans, 2008). Social fluency is deeply entangled with parents’ educational background, cultural capital, gender and emotional vulnerability (Russo et al., 2023). Parents with lower levels of formal education or cultural familiarity with medical institutions may find themselves at a disadvantage, struggling to engage meaningfully in discussions about their child’s care. The risk is that communication assumes a vertical and paternalistic form, in which parents are infantilised or, even unintentionally, excluded from a full understanding of the care process. Such asymmetries are not limited to the hospitalisation period. Rather, they tend to persist in the post-discharge phase, shaping families’ ongoing relationships with healthcare services, limiting their ability to act as informed caregivers (Silverman, 1987).

In this scenario, communicative competence is not equally distributed but is structurally mediated by broader axes of social inequality. These include not only education and class, but also emotional literacy, gendered expectations, and institutional norms of interaction (Bourdieu, 1986; Arendell, 2000; Jackson and Mannix, 2004; Lundqvist et al., 2007). Thus, what might appear as a lack of understanding or passivity on the part of parents is often a reflection of the institutional and symbolic asymmetries that define the NICU environment.

A second area of scholarly inquiry concerns the growing impact of digital technologies, particularly the Internet, on the relational dynamics between parents and healthcare professionals. The increasing accessibility of online sources, including medical or pseudo-medical information websites, discussion forums, and social media platforms, has profoundly altered the traditional asymmetry of knowledge and authority in clinical encounters. Parents are no longer mere passive recipients of professional expertise; rather, they are increasingly positioned as active interlocutors, often equipped with self-acquired knowledge that may challenge or reframe the authority of clinical professionals (Hardey, 2001; Lupton, 2012). As Evans (2008) has argued, this phenomenon does not necessarily translate into a democratisation of medical knowledge. Instead, it often results in heightened communicative tensions, as parents and professionals navigate the epistemic friction generated by decontextualised or misunderstood information gathered online. Parents may feel empowered to question or even contest medical decisions, whilst healthcare providers may perceive such participation as undermining their professional legitimacy and expertise (Henwood et al., 2003; Johnson, 2014). The consequences of this shift are inherently ambivalent. Digital technologies facilitate information access and foster parental empowerment; conversely, they increase the risk of misunderstanding, mistrust, and epistemic conflict (Mesman, 2020).

Recent studies have shown that digital platforms are also employed to build informal support networks among parents. Within these networks, emotions, advice, and experiential knowledge circulate, but so too does misinformation (Jiménez-Palomares et al., 2021; O’Connor and Madge, 2004). Further insight is provided by Cossetta and Caliandro (2013), who explore how mothers narrate their experiences of motherhood within online environments such as forums, blogs and social media. Their netnographic study of over 13,000 online conversations reveals that these digital narratives serve not only as spaces of reassurance and community-building but also as vehicles for challenging dominant biomedical framings of pregnancy and motherhood, valorising lay knowledge and resisting over-medicalisation. These expressive forms complicate the notion of the “informed parent” by foregrounding the affective and symbolic dimensions of digital health participation.

In this emerging communicative ecology, healthcare authority can no longer be taken for granted. It must instead be continuously negotiated, renegotiated, and co-constructed within relational and discursive frameworks that include both professional and lay forms of knowledge (Pols, 2013; Nettleton, 2004). Healthcare professionals are increasingly required to adopt adaptive strategies that safeguard communicative effectiveness and parental well-being without compromising the quality of care. These strategies involve adopting more accessible and inclusive language, offering emotional support, and engaging intermediary figures—such as psychologists, socio-health professionals and volunteers—who help mediate and enhance clinical communication (Gläser and Laudel, 2009). In certain clinical contexts, there is a discernible shift toward balancing scientific authority with a dialogical and pedagogical approach—one that acknowledges not only parents’ knowledge but also their emotional states, cultural backgrounds, and expectations (Lundqvist et al., 2007; Levetown, 2008; Russo and Decataldo, 2024).

Recent literature suggests that effective communication in NICUs cannot be reduced to the transmission of technical information. Rather, it must be reconfigured as a relational form of mediation, one that integrates multiple knowledge systems, manages clinical uncertainty, and contains parental distress (Franck and O'Brien, 2019; Mesman, 2014; Turner, 2006). International health institutions such as the World Health Organization (2020) and National Health and Medical Research Council (2000) have emphasised that communication should be understood as a core dimension of care quality, not as an optional or ancillary element. Crucially, the quality of communication has been shown to influence not only family satisfaction, but also the long-term clinical and psychosocial outcomes for the neonate (O’Brien et al., 2013; Wigert et al., 2014).

## 3 Materials and methods

This paper has three different aims: (1) analyse structural barriers, that impede effective communication between NICU staff and parents of pre-term children; (2) examine how digital technologies, especially the Internet, shape health staff and parents interactions and how it reconfigures power dynamics in clinical communication, and (3) investigate how healthcare professionals manage communication and which adaptive strategies they deploy to tackle the problems at hand.

To achieve these objectives, we draw on the qualitative components of the Italian PRIN (Projects of Relevant National Interest) project e-ParWelB, which employs two main methods, expert interviews and focused ethnography. These approaches enable us to examine the NICU environment from two complementary perspectives: the external researcher’s analytical lens and the lived experience of those working daily in the ward. For this paper, we focus primarily on the staff’s perspective, as their extensive firsthand experience in communicating with parents is central to our analysis. Ethnographic observations serve as a complementary source, enriching the interviews by grounding them in the lived reality of the NICU. This engagement with the wards offers insight into professionals’ daily work and the real-life dynamics of their evolving relationships with parents.

One of the authors of this paper personally conducted in-presence all the 76 semi-structured expert interviews and ethnographic observations. This allowed to avoid the effort and (potential misunderstandings) of training external interviewers; as a member of the research team, the interviewer was involved in conceptualizing the research problems, cognitive objectives, and theoretical and methodological framework, thereby possessing deep knowledge of the topic organised around four main domains. Consequently, the interview guide was outlined as follows: the first set of questions invited staff to introduce themselves and reflect on their professional experience at the NICU. Serving as an icebreaker, this section helped ease interviewees into a narrative mode whilst allowing space for unexpected insights. The second, and most central to this paper, focused on how relationships with parents are built and sustained. The third explored decision-making processes within the NICU and how different professional roles interact, shedding light on when staff consider it appropriate to involve parents. Finally, the fourth topic addressed staff perceptions of technology and its influence on parental behaviour.

Our sampling strategy was grounded in the principle of maximum variation (Flick, 2006), which guided the inclusion of a diverse range of professional roles and experiences within the NICU context. The selection of interviewees reflected our comprehensive stance on expertise not limited to formal, codified knowledge, but encompassing the embodied, situated understanding developed through everyday practice. Drawing on Gläser and Laudel’s (2009) framework, we conceptualise expertise as a socially embedded and evolving process, shaped by interaction, reflection and responsiveness to context. In this sense, we regard staff—such as neonatologists, nurses, and other health professionals—as experts not only by their formal accreditation but also from the practical knowledge cultivated through sustained engagement with preterm infants and their families. Their expertise is often “situated” and co-constructed through lived, context-specific experiences (Rashid et al., 2019). This view aligns with Collins and Evans’ (2007) concept of “interactional expertise,” which posits that meaningful knowledge arises from immersion in a community’s social and communicative practices, even beyond one’s formal disciplinary boundaries.

In total, we interviewed 76 NICU professionals, 67 women and 9 men. Only one interviewee was not of Italian origin. Regarding seniority, among the 32 doctors interviewed, 25% (*N* = 8) were doctors in specialist training, as defined by their contractual and professional status. For the other professional categories, levels of experience were not formally classified; however, indications emerging from the interviews allowed us to broadly infer their experience, which was taken into account in the analysis. The participants were recruited during the ethnographic observations. Overall, most of the potential participants accepted to be interviewed, even if it sometimes required gaining the general trust of the ward after a few days of observation. The ones who declined (*N* = 9) motivated their decision due to a personal desire to avoid being recorded. In those cases the researcher was allowed to take notes during informal conversations which do not qualify as an interview (and therefore cannot be quoted in verbatim). None of the professionals decided to drop out of the research. The total number of interviews was not predetermined but rather decided when the researcher felt the field was saturated. At the beginning of each field the researcher’s priority was to maximize the number of interviews. Only on the last days of the field, when the researcher felt that thematic saturation had been reached, requests for interviews with experts were stopped.

The Table 1 summarises the distribution of interviewees by professional role and hospital.

**Table 1:** Interviews distribution by hospital and professional role.

Hospitals	Professional roles							Total
	Medics	Nurses	Psychologists	Physiotherapists	Healthcare assistants	Volunteers	Obstetricians	
Yellow hospital	8	5	1	1	1	1	0	17
Blue hospital	5	9	1	0	2	0	1	17
Red hospital	10	9	1	0	2	0	0	22
Green hospital	9	8	1	0	1	0	0	19
Total	32	31	4	1	6	1	1	76

The fieldwork was conducted in four Italian hospitals: Yellow, Blue, and Green, located in the northern regions, and Red, situated in the “South and Islands” macro-area. All hospitals belong to the public healthcare system; however, Green is a privately managed institution with public funding and accreditation. To ensure anonymity, each hospital was assigned a pseudonym, as shown in Table 1. In the text, direct quotations are attributed using the interviewee’s role, interview number, and anonymised hospital name. We carefully excluded any excerpts that might reveal the hospital’s identity. Whilst this limits our ability to discuss certain institutional specificities, it ensures the privacy of participants. All NICUs are physically organised according to similar architectural features: every partner NICU is formed by two or three open spaces: an intensive therapy room, a sub-intensive therapy room and a pre-discharge room. In smaller wards, the former two spaces are usually located in the same room. Regarding the main clinical practices, all NICUs apply similar methods, which include a first round of briefings at a medical station, a space where the staff gathered separately from the rest of the ward and take clinical decision on patients, and subsequently, a bedside tour of the ward, which may engage with parents present in the NICU at that moment.

Ethical approval was obtained from the ethics committees of the partner universities of the e-ParWelB project (University of Milano-Bicocca and Catholic University of the Sacred Heart of Milan), and subsequently from the four territorial ethics committees affiliated with the hospitals involved in the research. Before every interview, each participant provided informed consent and authorisation for data processing by signing two documents approved by the ethics and data protection offices of all institutions involved, in full compliance with GDPR.

To strengthen the reliability of the data, we integrated interview material with ethnographic observations, which offered contextual insight and helped cross-check the narratives shared by participants. We conducted four different ethnographic fields, one for each hospital. Each field lasted for about 1 week, according to the principles of focused ethnography. Contrary to traditional observation, focused ethnography (Knoblauch, 2005; Wall, 2014) is based on brief and intensive observations to analyse well-defined phenomena within a specific context. This approach requires a redefinition of the ethnographer’s role, departing from traditional ethnographic models. Rather than entering the field as a blank slate, the researcher must possess a solid theoretical grounding in the relevant domain. Unlike classical ethnography, focused ethnography limits immersive engagement due to ethical and practical constraints. In clinical settings, for example, direct involvement in medical procedures is both inappropriate and unfeasible, positioning the ethnographer in a peripheral, observational role. Whilst these features may create problems in the study of wide informal networks, they align particularly well with medical research contexts, where interactions are often brief, structured, and protocol-driven. Its methodological relevance is supported by empirical work in clinical environments (Conte et al., 2015; Chopra et al., 2018). As we draw mainly upon interviews, we decided to value the point of view of the participants quoting some verbatim from the interviews. The ethnographic notes are not directly quoted but helped to support and validate our final data interpretations. All interview transcripts presented in this work were translated from Italian to English, with careful attention to preserving the original meaning.

### 3.1 Analysis strategies

The overall textual material was organised and analysed through a concept-driven coding strategy around the 3 research aims stated in the introduction paragraph, with NVIVO 15. A total of 76 interviews were transcribed and coded. The coding process was structured around a set of main thematic nodes that were defined *a priori* by the researchers in alignment with the research aims and applied deductively to the interview data. These included:

- Authority-related issues: when it is employed by the staff and when it is challenged by parents.
- Structural barriers: migrant background, vertical and horizontal cultural barriers, emotional barriers, gender barriers.
- Technology-related issues: the Internet, WhatsApp and other chatrooms.
- Staff behavioural strategies: pedagogical approach, emotional support, infantilization.

Each of these nodes was further articulated into specific sub-codes, developed iteratively during the analysis. In total, 15 sub-nodes were generated, with 514 references coded across the dataset. The table below presents the codes grouped by thematic family, along with the number of references and the number of interviews in which each code appears (Table 2).

**Table 2:** Overview of main thematic nodes, related sub-nodes, and their occurrence across interviews.

Thematic parent nodes	Sub-nodes	Interviews		References	
		*N*	%	*N*	%
Authority	Authority challenge	24	31.6%	44	9.4%
	Authority deployment	20	26.3%	32	6.9%
	Authority obeisance	5	6.6%	5	1.1%
Structural barriers	Educational barriers—horizontal	14	18.4%	17	3.6%
	Educational barriers—vertical	18	23.7%	20	4.3%
	Emotional barriers	30	39.5%	41	8.8%
	Ethnic barriers	18	23.7%	25	5.4%
	Gender barriers—mothers	4	5.3%	5	1.1%
	Gender barriers—fathers	3	3.9%	5	1.1%
Technology	Doctor google	16	21.1%	18	3.9%
	WhatsApp/peer group	21	27.6%	28	6.0%
Staff communication and behavioural strategies	Closeness building	46	60.5%	89	19.1%
	Emotional support	34	44.7%	48	10.3%
	Pedagogical approach	32	42.1%	53	11.4%
	Parents’ infantilisation	23	30.3%	36	7.7%

The most frequently referenced codes concern *Staff communication strategies*, particularly *closeness building* (89 references) and *emotional support* (48), followed by themes related to the *pedagogical approach* (53) and *parents’ infantilisation* (36). Codes related to *Authority* and *Structural barriers* were also widely represented, with multiple sub-nodes distributed across a substantial number of interviews. In contrast, gender-specific barriers appeared less frequently, suggesting either lower salience or less direct expression by participants.

Overall, the thematic node *Staff communication and behavioural strategies* cuts across all the dimensions explored in this study. This is reflected in the high number of references and interviews associated with the codes within this family. In contrast, the remaining three parent nodes are each primarily aligned with one of the study’s three research objectives: *Structural barriers* addresses the first one, *Technology* corresponds to the second, and *Authority* is predominantly linked to the third and final research aim.

## 4 Results

We present our research results following the three different research aims exposed at the beginning of the paper. The results are organised around four main thematic nodes (see Table 2) that mirror the main analytical focuses of the study. The first thematic node, *Structural Barriers*, encompasses the material and symbolic obstacles that hinder effective communication between healthcare staff and parents. These include linguistic and cultural divides, hierarchical dynamics across professional roles and emotional factors that influence openness and mutual understanding. The second node, *Technology*, explores how digital tools and online resources, ranging from *Doctor Google* to *WhatsApp* peer groups, mediate the relationship between parents and professionals. The third node, *Authority*, addresses the multifaceted ways in which professional authority is asserted, challenged and redefined in everyday interactions. This theme captures both overt and subtle negotiations of power, including instances where staff authority is contested, strategically deployed, or met with parental compliance. Finally, the theme labelled *Staff Communication and Behavioural Strategies* focuses on the adaptive practices clinicians employ to manage these complex interactions. These include efforts to build closeness and emotional support, the adoption of pedagogical approaches to enhance parental understanding, and strategies aimed at maintaining authority whilst avoiding the infantilisation of parents.

### 4.1 Barriers to effective communication: socio-cultural, educational, and structural factors

The first objective focuses on identifying the barriers, mainly linked to parents’ social backgrounds, that hinder effective communication with healthcare staff. One of the most reported issues is the difficulty in interacting with parents from migrant backgrounds. As hypothesised by Evans (2008), this situation creates two different and consequential problems: firstly, difficulties in communication with those who do not have a fluent mastery of the Italian language, and secondly, once the first problem is overcome, different culturally coded visions regarding parental roles and obligations.

*We have a lot of difficulties with foreigners because we never know what they think. Partly due to linguistic barriers, but also when they speak Italian. They have a different perspective on life.* (Medic 8, Yellow Hospital)

*During urgency it may be a problem having a family from Africa. We try to speak English and find out what language they understand in order to help each other. It may also be a problem with the socio-cultural situation; I mean, these families have different expectations regarding how to relate to pain and illness. Their relationship with newborns is different from what we expect.* (Nurse 2, Blue Hospital)

In these moments of incommunicability, especially during emergencies, healthcare staff face significant challenges in carrying out their work. The lack of mutual understanding often leads them to fill cognitive gaps with overly simplistic or exaggerated assumptions about parents’ behaviours.

*It is a cultural situation. If they give birth to preterm children, it is difficult for them to accept it because in their culture these children tend to die.* (Nurse 4, Red Hospital)

Among the different possible backgrounds, physicians seem to be particularly at odds with parents of Middle-Eastern or North-African origins, especially if they perceive said parents as Muslim. Specifically, they tend to consider the Muslim religion as an inherently degrading situation for mothers who are perceived as victims of their husbands and lacking any form of agency.

*It depends on the ethnic origins. I speak about the Muslim religion; you can see that the father acts as a filter, and the mother is isolated. […] You only speak with the father, it is very difficult to find an emancipated woman from North Africa.* (Medic 3, Blue Hospital)

*Most of these arrogant fathers are non-EU, because, we know very well, in their culture, women should not do anything. They don’t want to have anything to do with women; a woman who wants to impose rules (a nurse or a neonatologist) is unthinkable.* (Nurse 7, Red Hospital)

Italian health care does not consistently foresee the presence of cultural mediators in the wards. When they are available, it is often thanks to the efforts of the management of specific wards. Until these professionals are a proper part of the wards’ organogram, migratory background-related issues will remain a structural part of NICUs. Every ward tries to somehow solve this gap by involving eventual relatives with higher proficiency in Italian. In some cases, these relatives are older children attending Italian school, who find themselves in the position of translating sensitive information concerning their siblings.

*A big problem is the lack of cultural mediators. […] At times the only way is to let a relative in. Last time it was an eleven-year-old brother who spoke good Italian since he was born here.* (Nurse 6, Red Hospital)

The lack of consistent presence of mediators worsens the job of the few professionals available, who risk being perceived as aliens and therefore being distrusted by both parents and staff.

*We had Albanian parents who asked to avoid the presence of the mediator. We understood each other with an awkward Italian because we had the impression that the mediator was taking some liberties in the translation.* (Obstetrician, Blue Hospital)

Another important barrier is represented by the cultural capital of parents. Contrary to previous literature on the topic (Schillinger et al., 2003), it appears that this dimension does not seem to work in a unidirectional way, as high and low levels of cultural capital present peculiar challenges for healthcare staff.

*Oftentimes, people without a degree may express themselves in a very basic way but are also more willing to learn. The graduate often says something like “Well, I am an engineer". It does not have any link to being a father; you don’t need physics to change a diaper. On the other hand, when we have a difficult diagnosis, it is more difficult to be understood by someone who interrupted their school progression.* (Nurse 2, Red Hospital)

Whilst parents with lower levels of cultural capital may be more inclined to accept staff guidance without feeling the need to fully understand every detail, at the same time, and in line with Schillinger et al. (2003) findings, they often struggle to grasp complex concepts and require special attention during explanations. Low educational attainment may create linguistic and symbolic barriers.

*You must speak dialect with some people because they are used to speaking only dialect.* (Nurse 4, Blue Hospital)

*We saw that parents with low socialisation manifested less satisfaction and interest towards psychological counselling. I reviewed my methods, and I understood that my approach did not connect with the cognitive instruments that those people employ in their lives. […] Who is not used to a certain level of complexity may have difficulties with my questions, and therefore I need to change my approach.* (Psychologist, Blue Hospital)

Conversely, highly educated parents often have both the cultural resources to understand medical information and the material means to remain present with their children. In these cases, the barrier seems to lie more in a lack of trust.

*When parents are in a delicate situation, especially if they have a higher educational level, they struggle to blindly trust us. That’s because they search medical information elsewhere and then try to argue with us, asking a lot of details. Afterwards, we have to find specific moments with them to try to rebuild some trust.* (Nurse 8, Green Hospital)

Healthcare professionals express particular concern about this trend. On one hand, they feel their authority is being challenged by parents perceived as arrogant or eager to “play medic” or “be the real doctor of their children.” On the other, they worry that whilst these parents may acquire some technical knowledge, especially about life-support machines, they often lack the expertise to place it within the broader context of complex therapy. This conclusion on the staff’s behalf is coherent with previous findings regarding parents’ understanding of NICU’s procedures (Montgomery, 2006).

This tension between “fear of losing status” and concern over potential parental errors is central to parent-staff relationships and will recur throughout our analysis.

*They want, at all costs, to stop being a parent and become, in a way, the doctor of their child; they want to be part of the decisions. One thing that I always say to them is that their child needs a parent, not another doctor. Instead, they then overfocus on some aspects of the clinical picture, maybe the ones they find more worrying. I also understand that having a child in intensive therapy is something worrying.* (Medic 7, Red Hospital)

*The ones with a degree are the kind of people who think they are someone important, and they are also the ones who read everything on Google.* (Nurse 4, Red Hospital)

Parents with technical backgrounds—engineers being a frequently cited example—tend to concentrate on understanding the machinery, a coping strategy that may inadvertently create emotional distance from their child.

*For example, the engineer looks at every curve, wants to know in a very cold, rational way. Still, he does not see the child, because he watches only the surroundings. He gets the monitor that malfunctions and all the rules, but he forgets why he is here.* (Medic 8, Yellow Hospital)

*There is the typical engineer dad who always looks at the monitors and never at the child.* (Nurse 3, Green Hospital)

This focus on technical data often serves as a refuge, allowing parents to avoid confronting the emotional uncertainty surrounding their child’s condition. However, as the excerpts above show, it can become a vicious cycle, as they tend to fixate on the most alarming numbers and trends. This issue introduces the last important barrier between healthcare staff and parents: the emotional one. Trauma appears to be something endemic and natural in the ward. The emotions spawned by a newborn’s hospitalisation are extremely difficult to handle. The staff understands this and tries to give parents their space.

*The preterm child who gets hospitalised is not the child whose parents imagined; it has nothing to do with the child they idealised. When something happens that interrupts what people were dreaming of, they may need some time to realise what is going on. Even if you give them time and space, they may struggle to accept reality, and interfacing with them is very difficult.* (Nurse 5, Blue Hospital)

Some of the staff members understand how parents may be jealous of the extended time they spend with their children. This is especially true for the nurses who are with the children for eight or twelve-hour shifts and are usually the ones who have to deal more with the human aspect of this line of work. They realise how the separation they are creating between children and parents, whilst necessary for infants’ salvation, is perceived as deeply unnatural.

*We are forgiven if we manage to rebuild the symbiotic relationship between mother and child, which has been abruptly interrupted. Still, I feel we are child abductors. We steal the child and we allow them to see it within a plastic box. Sometimes we let them caress or hold their children, but I still feel like a robber.* (Nurse 6, Red Hospital)

This emotional distress directly impacts the effectiveness of medical communication. As registered by previous findings (O'Rourke et al., 2019), some parents are simply not ready to listen and elaborate on information. Health care staff tend to be aware of that and strategise their communications accordingly.

*Communication may even be effective from a strictly methodological point of view, but you have to be careful about the moment. There is always an emotional baggage which intervenes, if the parents are not ready or able to accept the news, you have to go back on it.* (Medic 2, Red Hospital)

Overall, the interviewees tend to believe that the situation is worsening, and parents are increasingly less able to deal with traumatic events. Older professionals maintain that they appear more fragile and lonelier than they used to be. According to the doctors’ and nurses’ interpretation, this event may have a demographic cause, as they feel new parents do not have extended families ready to support them both materially and emotionally.

*There is less familial support. Maybe the families are distant or maybe they have difficult situations, but I feel this is a general truth, not only a NICU situation.* (Medic 7, Red Hospital)

*Across the years, my perception is that parents are becoming frailer. There are more frail parents. They tend to be more anxious compared to the past. They ask for much more information, and they keep very insistently checking everything on the Internet.* (Medic 5, Red Hospital)

As shown in this last excerpt, another aspect that may make parents more anxious is the rise of Internet-related technologies. Each year, new software increases the potential communicative affordances available, and more and more websites provide unchecked medical-related information.

### 4.2 The role of digital technologies in shaping staff-parent interactions

Digital technologies, particularly the Internet, influence interactions between health staff and parents, reshaping power dynamics in clinical communication. Our findings suggest that these dynamics are primarily influenced by two key affordances: greater access to medical information and the simplification of interpersonal communication.

The rise of widespread availability of medical or pseudo-medical information on the Internet is radically changing the relationship between parents and healthcare staff around two main dimensions. The first is the power balance within the ward. According to previous studies (Henwood et al., 2003; Johnson, 2014), as the information becomes more accessible, the power of the old keepers of the lore wanes. Therefore, doctors and nurses express concern about losing influence over parents, often describing these changing behaviours with frustration or irritation. They often employ the sneering expression “they have asked doctor Google” to refer to parents who look on the Internet about what is happening to their children. They define them as arrogant, and they rant about the fact that they make their job impossible.

*There are a lot of rude parents who constantly try to diminish your job. Some do it out of ignorance, and those who do it out of arrogance. There are those parents who get a degree on Google and, after a month here, pretend to tell you what you need to do. It is completely impossible to build a collaborative relationship with these parents.* (Nurse 3, Red Hospital)

*There is the Google expert parent who tells you, even before you start speaking, that he has already understood everything, that he knows everything. These are the things that make me really mad. I really feel the jugular pulsing. Therefore, I count up to ten and then I start again as if he had not told me anything.* (Medic 10, Red Hospital)

Historically, healthcare professionals and especially physicians have occupied a highly privileged social position. Institutionalised medical violence has been a part of modern medicine since its inception, yet only recently has it become a sustained object of inquiry, both as a systemic dynamic (Farmer et al., 2016; Shapiro, 2018) and as a tool of control over marginalised bodies (Rojas Durazo, 2016; Malatino, 2019). However, whilst increased access to information allows parents to better understand their child’s condition, they often lack the medical expertise to interpret it accurately.

As it often surfaces in the ethnographic notes, the abundance of information complicates communication between staff and parents: whilst parents feel more empowered, they still lack the expertise to make informed decisions about therapy. As a result, healthcare professionals are concerned not only about their shifting authority, but also about the practical impact of this trend on their work and on parental behaviour.

Another problematic aspect of unsupervised information availability is represented by the fact that, according to staff members, the Internet increases parents’ levels of anxiety during an already difficult moment. They tend to give much weight to the risks and potential negative outcomes since they do not properly understand their children’s actual medical situation.

*They tend to go and see mainly the negative stuff, never the positive one. They focus on the worst possible outcomes for a child hospitalised in NICU.* (Nurse 5, Yellow Hospital)

*The worst thing is the fact that they go on the Internet and they fill their heads with all the negative aspects, never the positive ones.* (Nurse 2, Green Hospital)

Overall, the internet seems to be filling a gap left by partially inadequate staff communication. Yet this shift often worsens the situation: parents become more stressed, and professionals face added strain in an already complex role. A significant portion of healthcare staff acknowledge the problem and recognise their responsibility in addressing it.

*What we say to them is usually to ask more questions, not to be embarrassed about asking the same thing several times, rather than looking blindly. Sometimes what they find is not true.* (Nurse 2, Blue Hospital)

*The Internet forces us to improve our translations of technical language because often parents are still not understanding us.* (Nurse 2, Green Hospital)

Another key aspect of digital technology in staff-parent relationships is its impact on interpersonal communication. Since the early 2010s, the rapid rise of messaging apps like WhatsApp and Telegram has made group chats a widespread social norm. Whilst peer support among parents already existed, these tools have greatly enhanced their ability to organise. Today, in nearly every NICU exists at least one spontaneously formed and informal parents’ group chat—sometimes even separate ones for intensive care, sub-intensive care, or pre-discharge rooms. Once again, the effects are a mixed bag. On the one hand, this helps parents support each other. It helps them realise that they are not alone, other people are living the same trauma, and that they can attempt to come through together. On the other hand, it makes the staff’s job more difficult and, at times, exacerbates the feeling of anxiety parents are experiencing.

*They do their groups, for better or worse. They buck up with each other, but at the same time, when there is a couple of parents who spread doubts about everything and everyone, other parents stop trusting you as well.* (Nurse 3, Green Hospital)

*My colleagues get angry when they write in the group something like - Your child is crying and the nurses are doing nothing- or something like that. They do not have the technical knowledge to understand why we do something or something else.* (Nurse 8, Green Hospital)

We find here a repetition of the previous impasse. Parents legitimately want more accountability on the staff’s behalf, but at the same time, do not know exactly how to evaluate nurses’ or medics’ work. The staff members get angry and frustrated because they feel spied on whilst they are working. Moreover, the older cohorts of healthcare staff are distressed by the fact that, whilst they know how to operate the NICU machinery, they are not digital natives. They see how these new technologies are making their work harder, and they do not know how to react.

*Well, my younger colleagues, people in their twenties or thirties, are digital natives; therefore, it is normal for them to navigate those environments. But for us who are in our sixties, we have to learn how to behave in these new situations.* (Nurse 8, Green Hospital)

Technology continues to pose complex challenges with no straightforward solutions. Yet some healthcare professionals recognise that addressing these issues could become an opportunity to rethink and improve problematic aspects of medical practice.

*What does this situation teach us? It teaches us that parents have needs, and we should work towards these needs. We should help them and work on peer groups. […] We should reflect on the fact that parents keep organising these group chats for a reason rather than simply state that chat groups are bad for them.* (Medic 6, Green Hospital)

*Rather than a group where they keep talking about all the bad stuff, it may be better if there existed an association also including other parents whose children finished their time in the hospital. It could be something a little more guided, structured, and it would probably help them more.* (Medic 7, Green Hospital)

Moderated group chats, where healthcare professionals can respond to parents’ concerns, alongside associations that bring together current and former patient families with medical staff, offer promising avenues for support. These initiatives not only address the emotional and informational needs of parents but also foster a more collaborative and compassionate care environment. By embracing these new forms of connection, healthcare institutions can help transform a source of tension into a shared space for resilience, trust, and collective learning.

### 4.3 Clinician strategies in high-uncertainty communication

We investigate how clinicians manage communication in uncertain clinical contexts, applying adaptive strategies to uphold their professional authority whilst addressing parental distress. Although medical-patient communication is increasingly taught in medicine and nursing courses, the main part of how to relate to patients is something that healthcare professionals have to learn by themselves. Two main perspectives emerge: some believe formal academic training is essential, whilst others see it as a skill that can only be learned through experience in the field. The metaphor of the tight-rope walker, always in the balance, is used by a nurse to describe the precariousness in managing parent-staff relationships.

*A little more training would be nice. We walk as tight-rope walkers. It is very easy to slip off on one side or the other, say the wrong thing at the wrong moment. […] I would like to have more instruments in my toolkit. I understand that this is not our calling, we are not psychologists. We are just in support roles, but still, we have to deal with parents' outbursts.* (Nurse 8, Green Hospital)

However, for others, the relationship with parents is seen as something that cannot be objectified, formalised, and therefore transmitted “from above,” but being a deeply contextual interaction, it can only be learned and negotiated on the field.

*It is all very subjective since we are talking about human relationships. It cannot be ruled by bases, infosheets and schedules. It is a relational thing, I may interact in a certain way, have a certain feeling and then the person after me destroys all the job I have done.* (Nurse 3, Yellow Hospital)

This self-taught system creates a situation where both the objectives to be met in communicating with the parents and how the relationship is built vary wildly.

Some professionals prioritise the child’s best interest above all, whilst others view the child and parents as a single “macro-patient” requiring holistic care. Each offered a slightly different perspective on managing relationships and setting boundaries with parents. A recurring issue was whether to share their private phone number, a gesture that symbolically and practically signals a deeper connection beyond formal roles. Many avoid it, often due to past experiences and the emotional burden it may bring, especially in cases with poor clinical outcomes.

*You inevitably develop a more, “intimate connection”, at least for me, since I have always worked in pediatrics and I saw a lot of oncological children, a lot of death. Personally I would never give my number to a parent or ask how the child is after a month. […] I always try to limit myself because I already took home too much burden which should have been left in the workplace. At some point you must stop.* (Nurse 4, Yellow Hospital)

Regardless of the level of intimacy beyond the workplace, almost all interviewees agree that it is important to nurture relationships with parents, who are experiencing trauma and are disoriented when entering the NICU for the first few times. Relationships are built especially through small, everyday gestures and interactions, so healthcare staff strive to make the NICU an environment as harmonious and welcoming as possible for parents and, in turn, for NICU workers.

*I try to put them at ease. […] try to comfort them with tea and biscuits. If you try to create some kind of harmony things go smoother for both them and us. Otherwise you get in difficult situations on several levels: physical, mental and maybe even legal.* (Nurse 2, Yellow Hospital)

*They often feel disoriented and alone. If they find someone who smiles at them and says something comforting, it may create a bond and you become a reference point.* (Health Care Worker 1, Yellow Hospital)

The ability to listen, quickly understand parents’ needs and know how to engage with them emerged in several interviews as essential for building trust, alliance and easing parental stress.

*By listening them, watching how they approach the child and their couple dynamics, you immediately understand what they need. I can tell you that if you do that you frame them after only one week and then you know how to proceed.* (Nurse 4, Green Hospital)

These skills help build stronger, more reciprocal relationships, even if parents often gravitate toward a specific nurse or doctor, typically the first one they meet or the one they feel most at ease with. That person becomes their main point of contact, the one they trust most. Such dynamics can function effectively even without developing into more personal relationships beyond the NICU.

*If you are listening to them it comes in a pretty natural way and you manage to build some sort of relationship of trust with them. You realize that when they arrive they say - It is so nice that you are here today, I am happy - and they tell you about the previous day when you did not see each other. […] Still I always keep some distance, without ever explicit it. I do not know how to explain it; I am a nurse and you are a parent and you can always talk to me while you are here.* (Nurse 3, Green Hospital)

As Gläser and Laudel (2009) note, the development of soft skills among clinicians can be seen as a response to the growing demands of their role, which increasingly requires balancing medical treatment with relational care. Whilst genuine connections do not always form, professionals recognise the importance of engaging with all parents, even those with whom a natural rapport is harder to establish.

*There are parents with whom you manage to immediately create harmony, those who need more time, those who never reach that level. […] The approach is to try being welcoming with everyone and then to keep insisting on the relationship until something slowly works.* (Nurse 8, Green Hospital)

Beyond managing daily routines and interactions, some interviews highlighted also more structured, formalised approaches to supporting parents, typically in emotionally charged moments such as discharge or after a loss. For example, nurses may prepare decorated booklets or memory boxes for bereaved families or provide booklets to guide parents through the transition home. These collective gestures are usually bottom-up initiatives, reflecting staff responses to perceived gaps in parental support. Their implementation often depends on team composition, with younger professionals generally more open to departing from established routines than their more experienced colleagues.

*We have a series of techniques for accompanying these parents: informative booklets, all the kits we build for those children who do not make it, memory-books for parents, boxes, … There is an extremely active participation from the staff. The boxes for example are bought raw and then hand painted by some colleagues. Those objects are very felt and I believe parents, even during tragic moments, perceive that we are working with care.* (Nurse 5, Green Hospital)

*We had some issues with discharge which needed to be dealt with. We talked to each other and concluded that we need to all give the same instructions so the parents could go home with a precise guide to follow. […] We are all young colleagues here now, there has been a very big turnover […]. Therefore we decided to try to catch this occasion and we created this booklet […] with a nursing perspective. Then we called the medics as well and we created something complete which can be useful to everyone.* (Nurse 8, Green Hospital)

Overall, many healthcare professionals acknowledge a noticeable improvement in the attention and care devoted to preterm parents compared to previous years. This attention to relational care is increasingly framed not as a matter of personal kindness, but as a core component of professional competence, an essential part of the specific skill set required to work in a NICU

*I have been working here for a lot of years and I saw a lot of improvement. We kept developing a lot of projects: breastfeeding, attachment, kangaroo care, in 25 years there has been more sensitisation, training and, […], kindness or availability. Ten years ago some parents could rightly be angry at us, not because we were not professional but because we did not have the right words, or no words at all, and therefore less empathy. Now when they arrive we always try to welcome them in an emphatic environment.* (Nurse 1, Yellow Hospital)

Even from the doctors’ perspective, an empathetic and supportive approach is crucial when interacting with parents. In sensitive cases, they adjust the balance between closeness and distance based on the newborn’s condition and their assessment of the parents’ emotional needs. This calibrated approach often serves a dual purpose: to provide emotional support to families under stress and to protect younger colleagues from potentially difficult interactions with those parents perceived as more demanding or at risk of conflict.

*The heaviness is […] the management of families who are understandably shaken. I realise I am not pleasant, I have never been, but I know I am emphatic, ask anyone. I understand swiftly when a couple is difficult and therefore I manage to safeguard my younger colleagues from insults and arguments; it is my role. I cuddle some parents and then, when the situation is smoother, they are entrusted to other colleagues.* (Medic 1, Green Hospital)

This resonates with the role of “emotional managers” in the NICU pointed out by Mesman (2014), as staff work to ease parental fears and maintain emotional stability in high-stress situations.

Whilst healthcare professionals increasingly recognise the needs of preterm parents, they also point to a growing, widespread distrust in the healthcare system. Some parents view staff as part of a system they consider unreliable, leading to a broader erosion of trust that starts with institutions and extends to individual professionals. As the nurse quoted below suggests, this delegitimisation of expertise is not solely driven by parents’ greater access to medical information.

*I am not sure whether it is information availability or a new way to perceive the health care workers or both. When I started here I believe that information was as available as now but it rarely happened that someone questioned us. Now some people are already convinced that the worker, medic or nurse is the same, is doing something against the patient. They are convinced that here we are all incompetent and we work sloppily because that is how the system functions. Therefore I would not know whether it is just information availability or a whole new perspective.* (Nurse 3, Red Hospital)

The challenge to medical authority often does not arise from specific mistakes or shortcomings by the staff, but is an expression of a pre-existing distrust and underlying suspicion that some parents carry toward the healthcare system. This challenge can manifest explicitly as active protest or a constant search for mistakes. Overall, this means that professional authority is no longer taken for granted but must be continually proven and legitimised to earn credibility.

*Nowadays more and more parents come to the NICU waiting for your mistake. You already know that you cannot connect with these people. They are the ones who try to film you with their phone. Once I was doing an echocardiogram and I saw one out of the corner of my eye and asked: “Mrs are you making me a video?” She paled and answered: “No, no, I would never!”. I was sure because I saw the moron’s reflection in the monitor and I told her: “You have to delete it right now […]. Regulations state that this is a criminal offense.” After this she toned it down a little bit.* (Medic 10, Red Hospital)

Furthermore, professionals note a shift in the way patients understand their role and claims: nowadays, parents are more aware of their rights, less subordinate to the healthcare system. Consequently, they assume a more active role, with less deference towards the medical authority, sometimes approaching it in a very demanding and assertive manner.

*Over the years I saw parents’ behaviour change towards us, nurses and doctors, but also towards children. Once parents' approach was always like: <<I apologise, can I…?>> and now they believe it is all due for them. It is everything like <<I want it like this, I am here, I have rights, I need to have this, I need to have that>>. It has changed a lot.* (Nurse 2, Yellow Hospital)

This growing confidence can be seen as a form of social fluency, i.e., the ability to understand and participate in the expert domain of neonatal care. In our case, such fluency is not always linked to educational background or cultural capital, as highlighted in the literature (Evans, 2008; Russo et al., 2023), but often develops through prolonged exposure and lived experience within the clinical environment. When parents spend extended time in the NICU due to their newborn’s critical condition, they become familiar with the unit and staff, gaining confidence over time. These situations often foster strong, meaningful relationships; yet this closeness can at times blur the boundary between involvement and intrusion, leading to role overlap and tensions with professional authority.

*After a while […] knowing the environment and what is going on and their way to ask questions changes.* (Medic 3, Yellow Hospital)

*They should trust us, which happens in the majority of cases, but sometimes not. These times are not pleasant […]. It mainly happens during long hospitalisations, the difficult ones. You can see that parents take more and more space. This is not always good because they start to get too much confidence when they should remain in their places.* (Nurse 2, Blue Hospital)

Healthcare staff’s responses to the more or less explicit delegitimisation of their professionalism and authority can take various forms. From a micro perspective, we have already noted how some older doctors tend to manage more complicated situations with conflict prevention and de-escalation strategies; other times, friction between staff and parents fails to be prevented. Broadening the perspective, a typical form of reappropriation of medical authority sometimes translates into parents’ limited involvement in therapeutic decisions: these choices are normalised and almost always explained by healthcare staff as a lack of expertise of parents; therefore, the only alternative for them is to comply with the decisions of the institutional actors.

*I believe that they are not very involved in our decisions but I believe it is not that necessary. I believe we are trying to make the best decision for the children therefore we mainly involve them in the sense that we explain the reasons for our choices. It is quite difficult for a parent to say <<No>>. We had clashes but usually we explain our choices because they are unqualified, they do not have medical knowledge. What could they discuss?* (Nurse 2, Blue Hospital)

In this regard, professionals express more nuanced positions. Nurses sometimes perceive that parental involvement is marginalised by medical decision-making, especially in some highly sensitive cases such as the one reported below. This illustrates a form of internal critique of medical authority and an engagement in boundary-work to negotiate their professional roles within hierarchical medical structures (Allen, 2000). Moreover, it reveals how, at least in extreme scenarios, some staff perceive parental involvement as ethically legitimate and appropriate, even if it is not always considered in medical decisions.

*We had this child on the deathbed, so it was a delicate situation. We kept going until the end of our forces, the parents were exhausted, we were exhausted, the child was not recovering. He was only kept alive by the machines and after months of hospitalisation parents asked: <<Please, stop.>> and the medics answered: <<No, we keep going.>>. It is an ethical controversy, I understand, but parents were ready. They were ready for serious, well-handled accompaniment, something which could have been managed with dignity. But no, we had to wait for a breakdown. Emergency, reanimation and we called the parents once the child was already dead. Parents at times are treated as 5 years old children.* (Nurse 3, Yellow hospital)

Meanwhile, others acknowledge a recent shift toward greater parental involvement in therapeutic decisions, though not without some reservations, often linked to concerns about parents’ emotional involvement and lack of clinical expertise.

Nurse*: They are informed and involved. It is something that changed over time, once the medic decided alone what to do, now it is not like that anymore.*

Interviewer*: Would you say it is a bad thing?*

Nurse*: At times yes, because everyone has different skills. It is true that if a parent does not want something you can not do it, but it is a vicious circle. Parents want to decide but to decide you need knowledge and sometimes emotive involvement make you say <<Do everything possible>>. It is not easy.* (Nurse 4, Red Hospital)

Still others offer a functional-organisational interpretation, highlighting the practical benefits of limited parental involvement. Whilst accepting the value of a therapeutic alliance with families, this perspective emphasises how nurses’ work becomes more efficient and less conflict-prone when they are not required to dedicate time to interacting with parents. Beyond logistical concerns, the nurse notes that parents’ marginal role in decision-making also serves institutional interests by streamlining work and preserving staff control.

*I like the fact there is not much interference from the parents. Our NICU is structured in order to have many spaces of complete autonomy from them and this helps avoiding many conflictual situations. […] The main issue is time. Giving parents time means that 80% of your worktime is used in explanations, collaborations, … I know that from a familial triad point of view it would be the best thing, but for us, the nurses, the fact that most of the time they are away allows us to manage time in a bearable way.* (Nurse 3, Red Hospital)

This example aligns with Arabiat et al. (2018) observation that such dynamics may result in apparent agreement masking a lack of true understanding. Moreover, what appears as limited involvement is often shaped by symbolic and institutional asymmetries (Bourdieu, 1986; Arendell, 2000; Jackson and Mannix, 2004; Lundqvist et al., 2007), which preserve professional authority whilst formally upholding ideals of collaboration.

Finally, in this ongoing process of (co)construction and (re)negotiation of medical authority, staff expectations on parents also play a significant role. The literature has already focused on parents’ position in the NICU, defining their role as hybrid (Mesman, 2008), in which they occupy a grey area where they are neither strictly speaking patients nor mere visitors. This explains the diverse and sometimes inconsistent ways in which healthcare professionals interact with and conceptualise their role. On the one hand, everyone recognises their fragility and traumatic condition, so they are regarded as pseudo-patients: similarly to newborns, the repertoire of words and imagery used to describe them evokes care and nurturing. On the other hand, since they are not actual patients, they are subjected to a wide set of expectations that often exceed those usually placed on a typical adult patient. These relate primarily to their active and continuous presence in the NICU alongside their children, their availability to learn caregiving tasks and their emotional self-regulation. In this sense, their participation is not only encouraged for its therapeutic value for the infant but also demanded as a kind of moral obligation. This attitude was observed during ethnographic observations, particularly in informal conversations among doctors. Thus, we argue that the expectation of presence can be read as a genuine recognition of the parent’s role in the care process, a form of inclusion rooted in family-centered care principles (Pettoello-Mantovani et al., 2009; Committee on Hospital Care and Institute for Patient- and Family-Centered Care, 2012; Davidson et al., 2017). However, it can also be framed as a reactive response to broader discourses on participation and patient empowerment: if parents claim greater involvement in decision-making, they are implicitly required to earn that right through constant presence and compliance with the norms of the NICU.

*We explain to them how important it is to stray close to their child. Sadly, we see that after the first phase in which the child needs to stabilize, once the danger is over parents stay here very little even if we are open 24/7.* (Nurse 1, Yellow Hospital)

*I mean mothers have maternity leave. If I were a mother during my leave I would always be here. […] I would expect for a parent to be here from 10 a.m. to 7 p.m. They do not pay for lunch in the canteen, they have free parking and a lot of other benefits, therefore you can really see that some are lazy. The ones who really believe in it are always here.* (Nurse 1, Blue Hospital)

## 5 Discussion and conclusions

The findings reveal a relational landscape in NICUs marked by structural asymmetries and shifting power dynamics. Communication between healthcare professionals and parents is often strained by symbolic and practical barriers, particularly in the case of migrant families. Language difficulties and differing cultural norms around illness and parenthood frequently hinder mutual understanding, whilst the absence of institutional mediators further exacerbates these gaps (Evans, 2008; Bourdieu, 1986). Parental education and cultural capital emerge as additional fault lines. Contrary to previous studies focused on the disadvantages faced by less-educated parents (Schillinger et al., 2003), our results show that different levels of cultural capital pose different challenges. Whilst parents with limited education may be more receptive, they often lack the tools to fully grasp complex clinical information. In contrast, highly educated parents may assert themselves more confidently, request detailed explanations, or challenge decisions. Yet this assertiveness does not always translate into genuine understanding: horizontal asymmetries, where parents have general technical competence but lack biomedical contextualization, can result in misaligned expectations or superficial agreement (Montgomery, 2006; Arabiat et al., 2018).

Emotional distress, mainly made up of grief, fear and a sense of alienation from the caregiving process, further shapes these interactions. As Mesman (2014) observes, healthcare professionals often act as “emotional managers,” working to contain parental anxiety whilst maintaining clinical routines. This emotional and relational labour is not ancillary but constitutes a core dimension of care quality and safety. The capacity of professionals to engage empathetically, sustain trust, and interpret emotional cues directly influences parental confidence, and the overall therapeutic climate within NICUs. Such emotions are not merely background conditions but active forces that determine the quality and continuity of communication. Addressing them requires practical emotional literacy among staff, including training in empathetic listening, recognition of emotional cues, and strategies for responding to parental distress without compromising clinical decision-making.

In this perspective, specific training programmes should aim to enhance professionals’ relational, emotional, and reflective competences, recognising that emotional attunement and relational sensitivity are clinical skills in their own right, as essential as technical expertise. The main objectives would be to improve communicative empathy, strengthen emotional literacy, and support the development of mediation and digital literacy skills, enabling staff to manage complexity and diversity within NICU settings.

Digital technologies, particularly the Internet and group chats, could intensify the tensions between parents and healthcare professionals. On one hand, access to online information empowers parents, allowing them to question medical authority and seek greater transparency. On the other hand, it introduces new epistemic conflicts: professionals report that self-acquired knowledge, often fragmented or decontextualised, undermines trust and complicates communication (Henwood et al., 2003; Johnson, 2014; Lupton, 2012). Similarly, informal peer networks, whilst offering emotional support, can amplify doubt and anxiety when left unsupervised.

It becomes essential to guide parents of preterm infants toward a conscious and informed use of the Internet. Parents could be provided with guidance materials—such as curated lists of trustworthy websites, short video tutorials, or brochures—co-produced by healthcare institutions, parent organizations and communication experts. This would encourage independent yet informed navigation of online health information, helping families distinguish between evidence-based content and unverified sources, supporting their digital autonomy whilst reinforcing the therapeutic alliance. Training programmes should therefore be situated within this evolving communicative ecosystem, where digital mediation plays a key role. The educational context must acknowledge these tensions, promoting both critical awareness of online information and the ethical use of digital tools in healthcare communication.

The inclusion of accessible, evidence-based digital resources designed for parents can enhance transparency and trust, ensuring that families feel supported in their search for knowledge without relying solely on clinicians for interpretive guidance.

At this intersection between empowerment and disinformation, digital technologies play an ambivalent role: they democratize access to knowledge but simultaneously erode the epistemic authority of professionals. Future research should explore how participatory or co-designed digital platforms could mediate this tension—creating trustworthy, context-aware informational ecosystems co-produced by parents, clinicians, and mediators. Such tools could integrate verified medical content with spaces for peer dialogue, enhancing digital literacy whilst preserving trust. The reconfiguration of communicative asymmetries has created an increasingly tense environment. Parents have access to vast amounts of information, which allows or deludes them to better understand what happens to their children. This perceived empowerment increases expectations for staff accountability (Pols, 2013; Nettleton, 2004), yet rarely corresponds to the level of knowledge needed to interpret complex clinical decisions. As a result, the gap between professional expertise and lay knowledge continues to shape staff-parent relationships and underpins many of the observed conflicts.

Accordingly, the training framework should also include structured reflection and evaluation, enabling participants to assess changes in their communication styles, emotional management, and digital engagement with families.

A promising line of inquiry concerns how digital technologies could be used to bridge rather than widen this gap—for instance, through interactive information portals, visual decision aids, or multilingual digital companions co-developed with families. These approaches align with the broader paradigm of co-design in healthcare communication (Lupton, 2018), emphasizing the active involvement of users in the creation of tools that reflect their real informational needs and emotional experiences. Integrating gender-aware communication strategies—such as recognizing mothers’ and fathers’ differing roles, schedules, and emotional needs—can make such tools more inclusive and effective.

These participatory design practices also represent an educational methodology in themselves, fostering collaborative learning between staff, mediators, and parents, and reinforcing an institutional culture of dialogue rather than hierarchy.

Frictions often emerge clearly when the gap between medical expertise and bottom-up knowledge, between expert and non-expert wisdom become apparent. Healthcare staff consistently affirm their commitment to the child’s well-being and to building constructive relationships with families. They acknowledge the trauma parents experience and make efforts to offer reassurance and emotional support. However, especially the doctors, when they come under direct or indirect pressure from parents, or when their authority is questioned, tend to retreat into the safety of their technical expertise. In doing so, they draw a clear boundary between what they are legitimised to do, namely “cure,” and what parents, lacking clinical training, should refrain from doing. Targeted training can help professionals to manage these relational tensions, offering tools for negotiation, boundary-setting, and collaborative problem-solving in emotionally charged contexts.

Attempts to mediate these tensions are often informal and highly dependent on individual attitudes, clinical context, or organisational culture. Rather than being embedded in institutional structures, such mediation relies on personal initiative and remains inconsistently supported. Ideally, these processes should be facilitated by third-party professionals trained in communication and conflict resolution. However, the chronic underfunding of the Italian healthcare system, alongside long-standing staffing shortages and structural neglect (France et al., 2005; Toth, 2016), severely limits the potential to institutionalise such roles. The result is often a polarisation between two contrasting and extreme positions: on one side, a clinical-technocratic model that defends professional authority and status; on the other, a growing demand from parents for emotional and decision-making involvement. Both positions reflect legitimate concerns: clinicians require autonomy to apply their expertise but also have to be transparent, accountable and engage families appropriately in the care process. However, the lack of time, resources and structured spaces for reflection and mediation tends to exacerbate the distance between these two positions, rather than fostering dialogue or integration. Thus, instead of enabling a shared understanding, the system often reinforces separation.

Recognising and institutionalising the relational and emotional dimension of professional practice is therefore fundamental. These skills—often developed informally and under pressure—should be formally valued, resourced, and integrated into performance assessment, supervision, and continuous education.

Embedding the proposed training programmes within institutional frameworks—through continuous professional development, interdisciplinary workshops, and inclusion of cultural mediators—would transform these individual efforts into structured practices of collaborative care.

The most immediate and impactful intervention would be to allocate more resources to the healthcare system, first allowing for an increase in staffing levels, particularly of nurses and physicians. In turn, this would help reduce the individual workload, making time and space for the relational dimension of care to unfold as a shared, intentional practice rather than a residual activity. Supporting professionals in their emotional well-being and reflective capacity is also crucial to sustain the quality of care and prevent compassion fatigue. Psychological support staff should also be strengthened, both for families and for professionals regularly exposed to emotional strain. Similarly, the presence of cultural and language mediators should be guaranteed, particularly in contexts of high ethnic and linguistic diversity.

Beyond individual roles, a structural rethinking of the parent–staff relationship is necessary. This could include targeted training for both professionals and families, as well as the creation of dedicated liaison roles, for instance non-clinical figures such as parent advocates, mediators, or psychosocial professionals. This new figure’s tasks might include providing regular updates, ensuring consistent communication, guiding parents through care pathways, and directing them to the appropriate personnel when needed.

Here, digital tools could act as intermediaries, providing asynchronous communication channels or structured feedback systems that facilitate mutual understanding outside high-pressure moments. Yet, this requires careful attention to design ethics, data privacy, and inclusion, to prevent such tools from reproducing the very inequalities they aim to mitigate. These considerations should be explicitly included in the educational modules, promoting awareness of ethical digital design and inclusive communication principles. Future studies might also investigate hybrid mediation models combining human and digital interaction—for example, apps co-managed by mediators and clinicians to coordinate information flows, track parental concerns, and support emotional self-regulation.

Our findings also highlight the potential of parent associations as valuable allies. When aligned with NICU staff, they offer unique forms of peer support, qualitatively different from that provided by medical professionals. These groups deserve institutional support and physical space within hospital settings. Many preterm parents report feeling truly understood only by those who have shared the same experience. The emotional and practical challenges of prematurity are largely invisible to the broader public, and this lack of societal awareness often deepens parents’ sense of isolation. Peer support thus becomes a vital space for recognition and mutual understanding.

Finally, promoting genuinely democratic forms of parental involvement in NICUs presupposes the active engagement of families in the care process (Mishler, 1984; Pols, 2013). However, this ideal is often undermined by structural inequalities—such as disparities in socioeconomic status, education, linguistic competence, or access to transportation and time off work—which can severely constrain the capacity of some parents to participate. These asymmetries result in uneven access to presence, voice, and influence within the unit. Addressing such disparities requires not only material interventions (e.g., financial support, flexible visiting policies) but also a broader cultural shift that recognises and accommodates diverse forms of parental engagement, without penalising those who are structurally less equipped to conform to ideal models of involvement.

Digital inclusion must be part of this shift: equitable access to devices, connectivity, and user-friendly platforms is essential if digital technologies are to serve as tools of empowerment rather than instruments of exclusion. Digital inclusion initiatives—such as the provision of free Wi-Fi within hospitals, loaned tablets, or technical assistance—could ensure that all parents, regardless of socioeconomic background, have equal access to trustworthy digital tools and online communication channels integrated into the care process.

Whilst this study offers valuable insights, it is important to consider certain methodological and contextual limitations when interpreting the findings. Although we interviewed a wide range of professionals, the absence of parents’ voices means the findings may reflect only one side of the relationship, particularly when regarding potential or actual conflict. Additionally, the sensitive nature of the topic and the legal vulnerability of healthcare workers raise the possibility of social desirability bias. Whilst we do not assume intentional distortion, healthcare professionals may, even unconsciously, frame their actions and decisions in a more favorable light, particularly when describing sensitive interactions with parents. Although we sought to mitigate this through the inclusion of ethnographic observations, such bias cannot be fully ruled out and should be meaningfully considered. In sum, the emotional and relational work of healthcare professionals constitutes a form of invisible expertise that sustains the very possibility of care. Recognising, training, and supporting this dimension is not only an ethical imperative but also a structural condition for high-quality, family-centered neonatal care.

## Data Availability

The datasets presented in this article are not readily available because our dataset, consisting of interview transcripts, is subject to restrictions imposed by the ethics committee of our University and those of partner hospitals and may only be used in anonymised form and not shared publicly, in order to protect participants’ privacy. Requests to access the datasets should be directed to Alessandra Decataldo (alessandra.decataldo@unimib.it).

## Interview guide

General considerations about the NICU and neonatology

- What do you think about your job?
- What led you to work in neonatology?
- What do you think are the main strengths of this NICU? And of NICUs in general?
- What do you think are the main problems of this NICU? And of NICUs in general?

Relationship with parents

- Can you tell me about a particularly positive or negative experience you have had with parents?
- How do you interact with parents? Do you think there are standard procedures, or does each parent require starting from scratch?
- How do you manage emotional distance in your relationship with parents? Do you find it difficult to maintain boundaries?
- Based on your experience, do you think it’s possible to identify “types” or “groups” of parents who show similar behaviours?
- Do you think parents visit this NICU often enough? Could you explain why?

Decision-making process

- How does the process work when you make decisions regarding the babies’ future? What are the determining factors?
- How do you feel when making these decisions?
- What is your relationship like with professionals from other disciplines who collaborate with you in the NICU?
- To what extent are parents involved in this process, and in what ways?

Relationship with technology

- What do you think is the role of technology in the NICU decision-making process?
- Has technological progress improved your work? How much has it changed since you started?
- Are you confident about the technological and medical advances expected in the coming years?
- Do you think technological progress can help the parents of preterm babies? If so, in what way?
